# Intestinal Neuronal Dysplasia Type B as Rare Organic Cause of Refractory Constipation in Adults: A Case Report and Literature Review

**DOI:** 10.3390/clinpract16050095

**Published:** 2026-05-20

**Authors:** Rosana Troskot Perić, Branko Bakula, Nensi Orlandini, Tomislav Pavlović, Mato Perić, Gabrijela Stanić

**Affiliations:** 1 Department of Gastroenterology and Hepatology, Clinical Hospital Sveti Duh, 10000 Zagreb, Croatia; 2 Faculty of Health Studies, University of Rijeka, 51000 Rijeka, Croatia; 3 Department of Surgery, Clinical Hospital Sveti Duh, 10000 Zagreb, Croatia; 4 St. Catherine Specialty Hospital, 10000 Zagreb, Croatia; 5 Faculty of Health Sciences, University of Split, 21000 Split, Croatia; 6 University of Applied Health Sciences Zagreb, 10000 Zagreb, Croatia; 7 University North, 48000 Koprivnica, Croatia; 8 Department of Pathology and Cytology, Clinical Hospital Sveti Duh, 10000 Zagreb, Croatia; 9 Faculty of Health Studies, Catholic University of Croatia, 10000 Zagreb, Croatia

**Keywords:** intestinal neuronal dysplasia, chronic constipation, Hirschsprung disease

## Abstract

Background: Intestinal neuronal dysplasia type B is an entity characterized by disturbances in the enteric plexus, it most often occurs in childhood and can appear as an isolated disease or in combination with other neuropathies. The main symptoms are chronic constipation and intestinal obstruction. Case presentation: This paper presents the diagnostic work-up of a 40-year-old patient who was referred to a tertiary center due to refractory chronic constipation. A diagnosis of intestinal neuronal dysplasia type B was made after a surgical procedure based on pathohistological findings. Discussion: In a further multi-year follow-up, the patient was symptom-free with regular bowel movements, with a significant quality of life improvement. Conclusion: This confirmed that the chosen type of surgical procedure was appropriate.

## 1. Introduction

According to the etiology, chronic constipation (CC) is divided into secondary constipation (organic diseases, endocrinological and metabolic causes, neurological and myogenic causes, drugs, anorectal diseases, psychological causes and others) and idiopathic chronic constipation (CIC) [1]. One of the causes of secondary chronic constipation can be intestinal neuronal dysplasia (IND), an enteric plexus disorder that can be an isolated condition or occur along with other neuropathies [2]. IND includes two subtypes: IND-A and IND-B. IND-A is extremely rare and is characterized by congenital aplasia or hypoplasia of sympathetic innervation. This type occurs in the neonatal period with symptoms such as diarrhea, gastrointestinal bleeding and acute intestinal obstruction. IND-B accounts for more than 95% of all IND cases and is characterized by the histological finding corresponding to hyperplasia of submucosal ganglion cells. This type can occur at any age, from a newborn to older adults. It is most commonly clinically manifested by chronic refractory constipation, but can also present with other clinical findings such as acute and chronic intestinal obstruction and necrotizing enterocolitis [2,3]. IND-B is a rare entity; the frequency is one case in every 7500 newborns [4]. It occurs most commonly in childhood, and most series of case reports are presented for that age, but it can also occur in adulthood. Additionally, in a clinical presentation, IND-B may mimic Hirschsprung’s disease (HD), which may influence further treatment decisions [2]. HD is a motor disorder of the intestine, caused by the failure of neural crest cells to migrate during intestinal development. This results in an aganglionic segment of the colon that cannot relax, causing functional obstruction. It is mostly diagnosed in neonatal periods; however, it can also be diagnosed later, even in adulthood. Clinically, it manifests as constipation, abdominal distension, and vomiting [5,6].

This paper presents a rare case of an adult patient with refractory chronic constipation caused by IND-B, with a review of recent literature, and with a differential diagnosis analysis of this entity.

## 2. Case Report

### 2.1. Disease Data

A 40-year-old patient was referred to the National Reference Center (NRC) for Functional Gastrointestinal Disorders of the Sveti Duh Clinical Hospital due to severe and refractory constipation. Since early childhood, the patient had constipation (<3 stools per week) that did not require medication or additional gastroenterological examinations. In a traffic accident, at the age of 18, he suffered a spinal injury, without fractures. Since then, he no longer had spontaneous bowel movements, but began taking laxatives as part of his therapy. Throughout his life, he had to change types of laxatives more regularly due to their loss of effectiveness, from stool softeners, osmotic laxatives, to contact laxatives. Approximately 10 years before reporting to the NRC, the patient noticed that the laxatives he had been using had become less effective, and began taking combinations of different types of laxatives with increasing doses. It is important to note that two years before reporting to the NRC, he had even noticed that a combination of laxatives was no longer effective, and the only thing that helped him was self-enema, which was performed once a week. The stools that he managed to induce with enemas were watery or softer. For the above reasons, the doctor referred him to a gastroenterologist at another institution. Despite the cleansing performed, it was not possible to perform a colonoscopy examination due to the stool formed in the colon lumen. Subsequently, the patient was referred to the NRC for further examination for the first time.

After admission to the NRC, during the physical examination of the patient, tenderness was found on deep palpation in the lower left abdomen, along with a painless mass, which was the size of a handball suprapubically. The body mass index (BMI) was normal (22.5 kg/m^2^), appetite was maintained, urination was without difficulty. The patient is a non-smoker, does not consume alcohol, and has no history of allergies.

**Figure 1:**
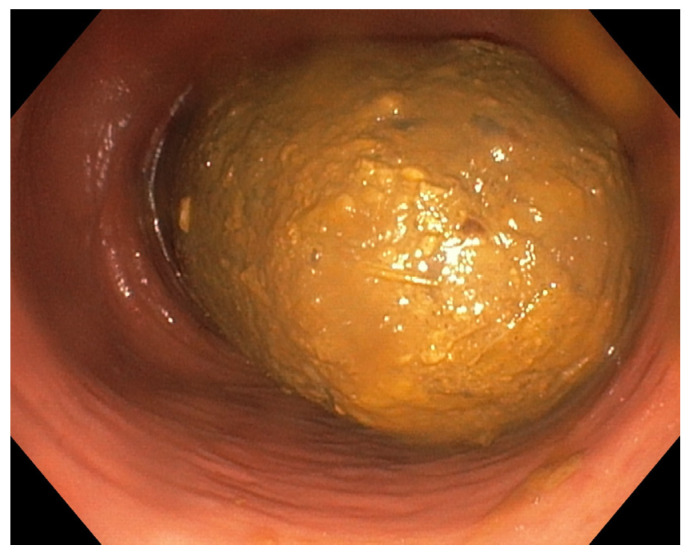
Colonoscopy with large fecal mass.

Upon examination, laboratory processing, abdominal ultrasound and endoscopic examinations were initially performed. The results of all laboratory findings (complete blood count, C-reactive protein, erythrocyte sedimentation rate, coagulation parameters, complete urine, liver enzymes, serum urea and creatinine, blood sugar, urates, electrolytes, complete lipidogram, proteinogram with electrophoresis and immunoelectrophoresis, serum iron, ferritin, thyroid-stimulating hormone and tumor markers) were normal. The abdominal ultrasound revealed marked flatulence with a paraumbilical reflective area and a wide distal acoustic shadow (intestinal coil appearance). The upper endoscopy showed reflux esophagitis grade A (LA classification) and hyperemia of the antropyloric region (pathohistological finding: chronic *Helicobacter pylori* negative gastritis). About 20 cm from the anocutaneous border was examined colonoscopically, it was not possible to pass more proximally due to the tamponade of the lumen with hard fecal masses (Figure 1).

**Figure 2:**
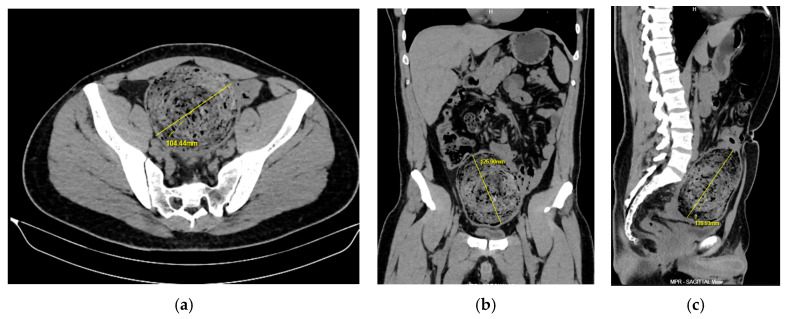
Computed tomography abdomen/pelvis in (**a**) axial plane, (**b**) coronal plane, (**c**) sagittal plane. Sigmoid colonic dilation with 11 cm large fecal mass.

Furthermore, the Multislice Computed Tomography (MSCT) of the abdomen showed coprostasis of the right colon and enlargement of the sigmoid part lumen with a diameter of 11 cm, which was tamponed with fecal mass (Figure 2a–c).

**Figure 3:**
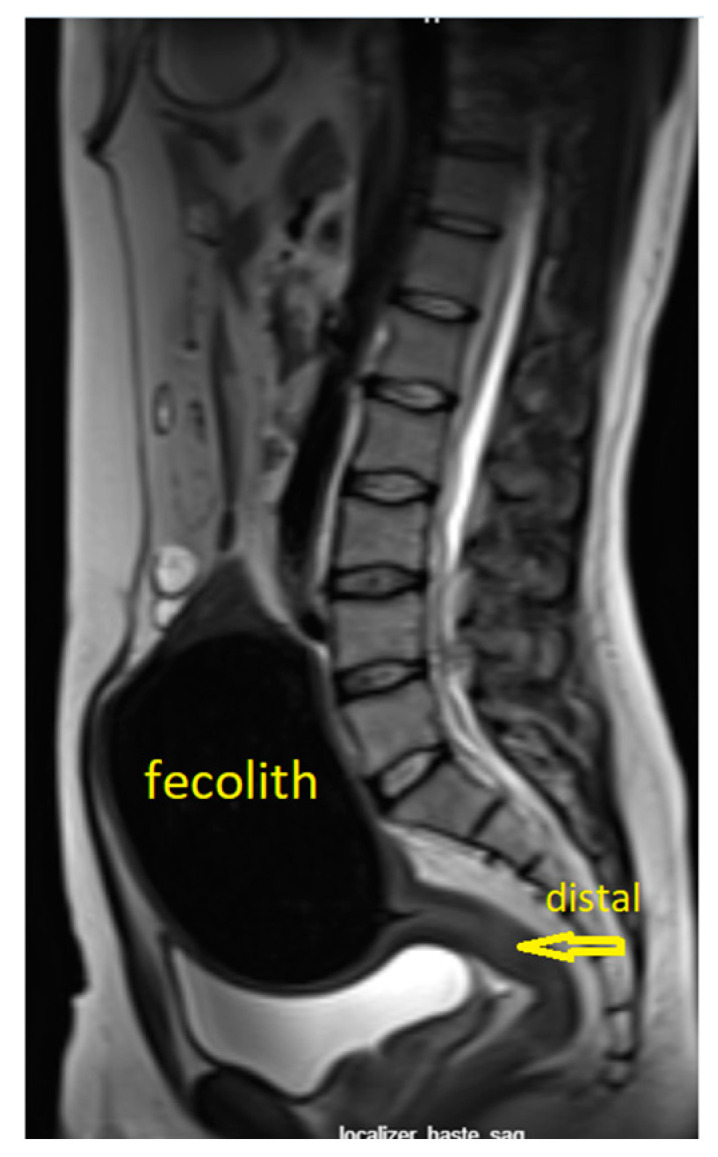
Magnetic Resonance Imaging enterography with large fecal mass at sigmoid colon.

The Magnetic Resonance Imaging (MRI) enterography showed an orderly haustrated intestine and a mildly dilated intestine proximal to the obstruction, but without signs of ileus, and an orderly part of the colon and rectum distal to the obstruction (Figure 3).

Additional specific examinations were performed of the gastrointestinal system using high-resolution anorectal manometry (HRAM) and colon transit time (CTT). A solid-state catheter was used for HRAM and the examination was performed according to a standardized testing protocol. The rectoanal inhibitory reflex (RAIR) was properly induced with an initial volume of 30 mL. The CTT revealed slow transit constipation: more than 5 days after the start of the examination, radiopaque markers were seen in the right colon and in the descending part of the colon. Medical examinations ruled out systemic and neurological diseases.

The patient was presented to the Multidisciplinary Team (MDT), where it was assessed that a de-impaction or endoscopic fragmentation of the stool would not be successful. There were no signs of ileus or other emergencies, but it was the surgeon’s assessment that operative treatment should be started due to the long mechanical load. Additionally, the patient was further motivated for operative treatment, due to long-lasting symptoms.

### 2.2. Surgical Findings

**Figure 4:**
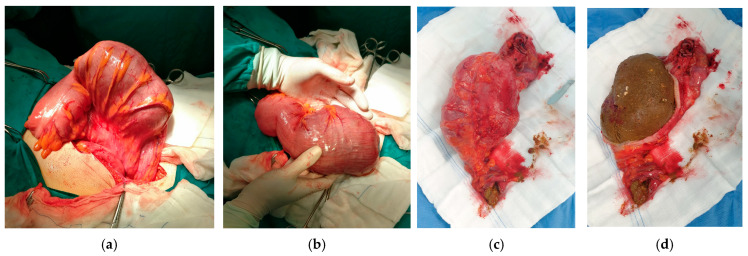
(**a**,**b**) Intraoperative finding of huge impacted fecolith in sigmoid colon. No signs found of colon ischemia, perforation or inflammation. Mild prestenotic colon dilatation found on short segment just proximal to fecolith, but colon without signs of ileus; (**c**) resected sigmoid colon segment showing normal caliber of colon distally and proximally to fecolith; (**d**) view of large impacted fecolith after opening of colon on side table.

Given the size of the process, an open surgical procedure was opted, and the abdomen was accessed via a midline laparotomy. Upon entering the abdomen, a huge round fecolith was found within the distal segment of the sigmoid colon, the size of a human fist (Figure 4a–d).

Intraoperatively, the entire colon was examined and assessed completely normal—vital, without any signs of ileus. A slight discrete prestenotic dilatation was observed on a shorter segment of a few centimeters just proximal to the fecolith. Given that no transition point was found and that it was a benign disease, a maximally sparing resection procedure was performed, resecting only the part of the intestine where the fecolith was located. Since the bowel appeared completely healthy macroscopically, without dilatation and properly toned, a primary establishment of continuity was made.

Postoperative recovery was normal: the patient first took liquid food, then soft food was continued, he passed stool after 3 days and had no problems with intake and absorption of food. The patient was discharged on the sixth postoperative day.

### 2.3. Pathohistological Findings

**Figure 5:**
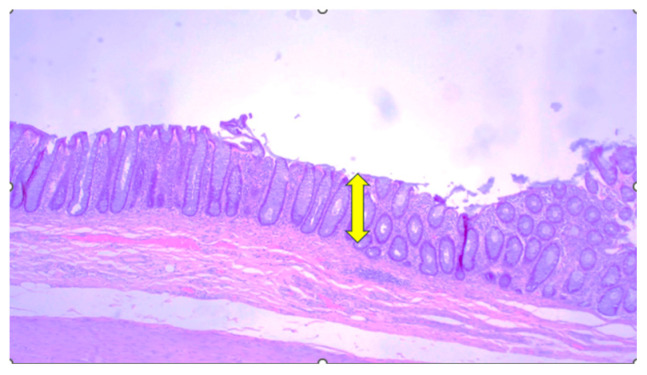
Histological findings (hematoxylin and eosin, 40×): thinned colonic mucosa (yellow arrow).

The Department of Pathology received a 25 cm resected colon with a markedly dilated lumen of 10 cm in length, which was tamponaded with fecal mass; the wall of the resected colon was markedly thinned. Furthermore, the histological findings showed a thinned colonic mucosa with preserved ganglia of the submucosal and myenteric plexus (Figure 5).

**Figure 6:**
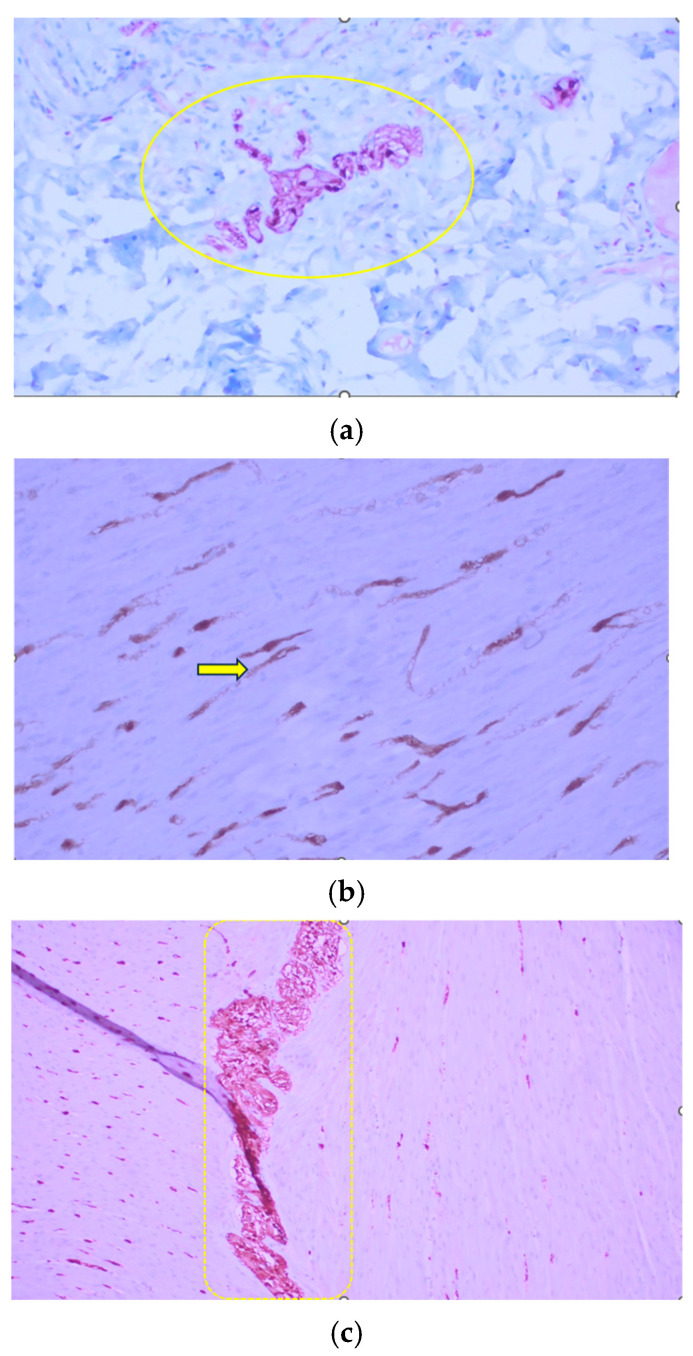
Immunohistochemical labeling with S100 protein (100×): (**a**) submucosal ganglion cells (ellipse), (**b**) nerve fiber (arrow), (**c**) myenteric plexus ganglion (rectangle).

**Figure 7:**
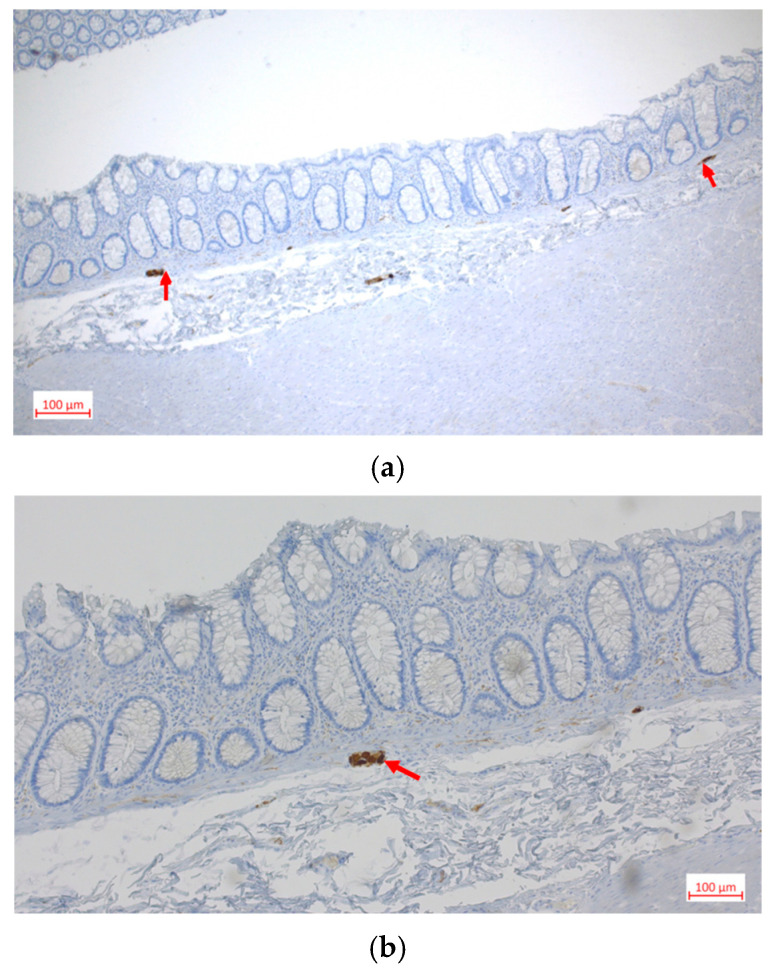
Immunohistochemical labeling with calretinin: (**a**) 40×, (**b**) 100×; heterotopic ganglia (red arrows).

The immunohistochemical evaluation included S100 protein and calretinin. The S100 protein highlighted neural structures within the enteric plexus, while calretinin was used to assess the presence of ganglion cells. Immunohistochemical labeling with S100 protein revealed more than eight ganglion cells in individual ganglia of the submucosal plexus with hypertrophic nerve fibers, as well as individual heterotopic neurons in the muscularis mucosa layer and hypertrophic nerve fibers in the muscularis layer (Figure 6a–c). Immunohistochemical staining with calretinin showed heterotopic ganglia in the muscularis mucosae (Figure 7a,b). Based on the histological and immunohistochemical findings, IND-B was suspected.

### 2.4. Follow-Up

After the operation, a control colonoscopy was performed, which was shown to be normal. Rectal biopsies were also taken, which did not contain a representative amount of submucosa for adequate interpretation.

During the 5-year follow-up period, the patient was free of postoperative complications or recurrent symptoms. Regular, spontaneous bowel movements (1–2× daily) were achieved, without the need for laxatives or enemas. The patient reports a high quality of life level and good functional status.

## 3. Discussion

This paper presents the case of a patient in adulthood, which clinically manifested as a severe and refractory form of chronic constipation. The patient had had problems with bowel movement since early childhood, even before the car accident, and it was considered that further worsening of the problems was not directly related to neurogenic bowel dysfunction. It is known that neurological causes such as spinal cord injuries, multiple sclerosis, Parkinson’s disease and others can lead to changes in bowel behavior, in the form of stool incontinence or constipation [7]. However, we cannot rule out that the trauma he was exposed to, both physically and psychologically (protracted stress), could have been a contributing cause to the further worsening of his symptoms.

According to the European Society of Neurogastroenterology and Motility guidelines on functional constipation in adults [8], patients with refractory CC should be referred to tertiary and reference centers for additional specific tests and a decision on the choice of further therapy. Regarding specific procedures, it is recommended to perform HRAM in order to rule out evacuation disorder (ED), which may be the cause of chronic constipation or may be present in combination with chronic constipation [8,9]. In addition, CTT should be used to determine whether it is slow transit or normal transit constipation [8].

According to the Frankfurt consensus, based on morphology, IND-B is characterized by hyperplasia of the submucosal nerve plexus [10]. For the sake of better diagnostic standardization, quantitative criteria related to counting the number of ganglion cells have been proposed: the presence of giant ganglia in the submucosa of the colon at more than 20%, which should contain more than eight neuronal cell bodies [11,12]. In our institution, S100 and calretinin represent the available immunohistochemical panel for the evaluation of enteric nervous system architecture during diagnosis, i.e., suspicion of IND. It is important to point out that acetylcholinesterase (AChE) histochemistry can represent an additional useful tool in the evaluation of suspected intestinal dysganglionosis [13]. In our case, these criteria were only partially met due to limited sampling and the absence of systematic quantitative and serial section evaluation. Following a comprehensive clinical evaluation, functional testing, and histopathological assessment, along with the exclusion of other chronic constipation causes, the findings were interpreted as suggestive of IND-B.

Nevertheless, the largest number of published works on IND-B refer to the inconsistencies that still exist in the pathohistological diagnostic criteria and methods used to define IND-B [14,15]. In clinical practice, this may mean that decisions on further treatment of the patient are not made depending on the presence or absence of pathohistological changes associated with IND-B. Therefore, IND-B was previously considered a pathohistological entity, but not a clinical entity. In a recently published work [16], 27 patients aged up to 15 with a histopathological diagnosis of IND-B, who had previously undergone colorectal resections, were included. The authors showed that, in these patients at the time of diagnosis, there was an association between clinical features (intestinal symptom index) and pathohistological changes that characterize IND-B. Even though there were some limitations to this work as it was a smaller case series and without a control group, the results of this study support the understanding of IND-B as an entity, that is, a disease.

Additionally, IND-B may have the same clinical presentation as HD and ‘allied disorders of Hirschsprung’s disease’. In addition to IND, these include disorders such as ganglionic immaturity, hypoganglionosis, megacystis microcolon intestinal hypoperistalsis syndrome, segmental dilatation, achalasia of the internal anal sphincter, and chronic idiopathic intestinal pseudo-obstruction, possibly posing a significant differential diagnostic problem [17]. IND-B can occur as an isolated condition or in combination with other enteric nervous system disorders such as HD. Some studies state that HD appears in combination with IND in 44% of patients with HD [18]; however, these results have not been confirmed in other studies [19,20]. Postoperative pull-through obstruction due to anatomical (mechanical or pathohistological) and physiological causes can occur in patients who have undergone surgery for HD. It is important to point out that IND belongs to the pathohistological causes that can correlate with HD and cause disturbances in intestinal motility, which can then lead to obstructive symptoms in these patients [21].

In accordance with the fact that IND-B can coexist with some other disorders of the enteric nervous system immaturity, the pathogenetic mechanisms of IND-B are not sufficiently clarified and there are several hypotheses that try to shed light on the possible causes [22]. One hypothesis is that IND is a neurological developmental defect, where genetic changes affect the embryonic development of the enteric neuronal system. Another hypothesis is that it is a secondary disorder, an adaptive response of the enteric nervous system to stimuli such as congenital intestinal obstructive factors and local inflammatory processes. However, the results of studies are contradictory, and the results of some studies performed on animal models are not reproducible [22].

In clinical practice, IND-B causes controversy, especially when it appears in adulthood or old age. In the study [23], a 71-year-old patient with long-term constipation since early youth was presented. The patient was hospitalized several times, and sigmoidectomy was performed. The diagnosis of IND-B was made only at an older age, when he was hospitalized due to intestinal obstruction, i.e., megacolon. The diagnosis was established on the basis of pathohistological findings and diagnostic criteria for IND. A similar clinical presentation was shown in our patient who also had long-term constipation that was not investigated. However, in our patient the gradual worsening of constipation over a number of years was more pronounced, and the patient no longer responded to conservative therapy. In both presented cases, it remains an open question whether long-term constipation could have led to pathohistological changes in the colon corresponding to IND or whether IND as an entity was the cause of chronic constipation.

A 2016 review of IND-B [2] listed several interesting cases of IND-B in adults that had been described so far. Some of the cases had symptoms since childhood, while others developed symptoms only in adulthood. Also, some of them had serious complications such as megacolon, chronic intestinal pseudo-obstruction, or intestinal infarction. IND-B was mostly localized in the distal part of the colon, but there were cases where the entire intestine was affected [23,24], or a rare localization in the ileum was described [25]. Also, the number of recently published papers on IND-B in adults is small and are individual case reports [26,27,28]. Masuda et al. [26] reported the case of a 36-year-old patient with long-standing refractory constipation who underwent total colectomy with ileorectal anastomosis using a modified single-incision laparoscopic surgery (SILS) technique. Hokama et al. [27] presented a case of a 26-year-old man with chronic constipation and acute massive volvulus of the sigmoid colon that was clearly seen on radiographs and MSCT of the abdomen. Initially, endoscopic decompression and detorsion were performed, but an elective total colectomy with ileorectal anastomosis was finally performed. In the work of Ren et al. [28] it was an unusual case of IND-B that manifested as a left psoas abscess in a 31-year-old man. Colonoscopy revealed stenosis of the terminal ileum, cecum, and the initial part of the ascending colon, which led to the placement of a temporary ileostomy, during which full-thickness colon wall biopsies were taken. In all the above case reports, the patients did not have any further problems after surgical procedures, which was also the case in our patient. Since our patient had a dilated sigmoid colon with a huge fecal mass in the lumen, we decided to perform a resection of the sigmoid colon, i.e., the dilated part, because the rest of the colon was normal on palpation and appearance.

Recently updated recommendations on the evaluation and treatment of refractory constipation [29] state that, after failure of non-pharmacological measures and pharmacological therapy, surgical treatment is a further option for these patients. Before surgical treatment of refractory constipation, it is necessary to confirm that it is slow transit constipation. The CTT results in our patient initially suggested slow transit constipation. However, this transit pattern was interpreted as secondary to distal functional obstruction due to long-term fecal impaction. In the review [30], the importance of caution in the interpretation of transit studies in the presence of significant mechanical impaction is emphasized.

It is important to note that due to the severe symptoms mentioned, the diagnosis of IND-B is mostly made after surgery based on histopathological findings. Depending on the clinical presentation and findings during the surgical procedure, the surgeon makes a decision on the type of surgical procedure: colectomy with ileoanal anastomosis, ileostomy, or resection of the affected intestinal segment. In the case of a patient with long-standing refractory constipation, Masuda et al. [26] decided to apply a modified single-incision laparoscopic surgery to minimize trauma and speed up the patient’s recovery. In our case report, based on clinical, imaging, and endoscopic findings, the operative approach was limited resection of the sigmoid colon containing a massive fecolith, resulting in excellent long-term improvement. Also, the results of other case reports mentioned above showed that patients had regular bowel movements and a good quality of life after surgery, regardless of the surgical technique [26,27,28].

## 4. Conclusions

IND-B represents the immaturity of the enteric nervous system and primarily affects the younger population, mainly children, but it can also occur in adulthood, even with the elderly, as confirmed by literature data. The main symptom is chronic constipation, which is why it is necessary to perform an extensive diagnostic workup to exclude numerous other causes of constipation. In our paper, the case of a 40-year-old patient is presented with severe and refractory chronic constipation. Due to the finding of a huge fecolith within the distal segment of the sigmoid colon, non-oncological resection of the affected part of the sigmoid colon was performed, with the establishment of continuity by creating a termino-terminal colorectal anastomosis. After clinical evaluation and histopathological assessment, and exclusion of other causes of chronic constipation, the findings were interpreted as suggestive of IND-B. A long-term follow-up period confirmed the excellent results: the patient became asymptomatic, with a fully established physiological bowel rhythm. This outcome serves as clinical confirmation that the choice of the type of surgical procedure was optimal for the patient.

## Data Availability

The original contributions presented in this study are included in the article.
